# Brazilian health professionals’ perception about the Baby-Led Weaning (BLW) method for complementary feeding: an exploratory study

**DOI:** 10.1590/1984-0462/2022/40/2020321

**Published:** 2021-10-04

**Authors:** Felipe Silva Neves, Bruna Miranda Romano, Angélica Atala Lombelo Campos, Camila Almeida Pavam, Renata Maria Souza Oliveira, Ana Paula Carlos Cândido, Michele Pereira

**Affiliations:** a Universidade Federal de Juiz de Fora, Juiz de Fora, MG, Brazil.

**Keywords:** Health personnel, Child, Infant, Weaning, Infant nutrition, Profissionais de saúde, Criança, Lactente, Desmame, Nutrição infantil

## Abstract

**Objective:**

To describe Brazilian health professionals’ perception about the Baby-Led Weaning (BLW) method use for complementary feeding.

**Methods:**

Cross-sectional, descriptive study including 458 health professionals graduated in Nursing, Speech Therapy, Medicine, Nutrition or Dentistry and working in Pediatrics, being directly or indirectly involved with pediatric nutrition. We used a convenience non-probability sampling. The questionnaire applied to participants addressed demographic characteristics, academic degree, workplace, knowledge about clinical practice and perceptions about the possible advantages of the BLW method.

**Results:**

Participants had a mean age of 34.5±8.5 years, 64.6% of them working in Southeast Brazil and 65.3% being nutritionists. Most participants reported being acquainted with the BLW method (82.0%). Regarding clinical practice, 38.3% mentioned having recommended the BLW some times, 37.5% often and 20.5% always. Most participants fully agreed that the BLW method could have advantages for babies, for example, having them more likely to share family meals, facilitating adaptation to food flavors and consistencies, enhancing chewing and favoring the development of motor skills. On the other hand, important disagreements were also expressed regarding the BLW convenience and the possibility to create less concerns or anxiety in parents.

**Conclusions:**

The BLW method reported as advantageous, but disagreements were also raised, probably because scientific evidences on the suject are scarse. Further investigation is needed so we can better understand the risks and benefits and health care professionals can feel effectively assisted to offer support and advice to parents and caretakers.

## Resumo

**Objetivo:**

Descrever as percepções de profissionais de saúde brasileiros acerca do método *baby-led weaning* (BLW) para alimentação complementar.

**Métodos:**

Estudo de corte transversal conduzido com 458 profissionais de saúde graduados em Enfermagem, Fonoaudiologia, Medicina, Nutrição ou Odontologia e que atuavam em pediatria, estando direta ou indiretamente envolvidos com nutrição infantil. A amostragem foi não probabilística, de conveniência. O questionário englobou características demográficas, titulação e local de trabalho dos participantes, além do conhecimento, da prática clínica e das percepções sobre possíveis vantagens do BLW.

**Resultados:**

Os profissionais de saúde tinham idade média de 34,5±8,5 anos, 64,6% atuavam no sudeste do Brasil e 65,3% eram nutricionistas. A maioria relatou conhecer o BLW (82,1%). Quanto à prática clínica, 38,3% mencionaram que costumavam recomendar o BLW às vezes, 37,5%, frequentemente, e 20,5%, sempre. Grande parte declarou que concordava totalmente que o método poderia trazer vantagens, tornando os bebês mais propensos a compartilharem refeições em família, facilitando a adaptação aos sabores e às consistências dos alimentos, potencializando a mastigação e favorecendo o desenvolvimento de habilidades motoras, porém manifestaram-se discordâncias importantes em relação à comodidade/conveniência e ao argumento de gerar menos preocupação ou ansiedade para os pais/cuidadores.

**Conclusões:**

Em geral, o BLW foi relatado como vantajoso, mas foram evidenciadas discordâncias, que são um provável reflexo da escassez de evidências científicas factuais sobre essa temática. São necessários mais estudos, para a melhor compressão de seus riscos e benefícios. Só assim os profissionais de saúde se sentirão efetivamente respaldados para fornecer suporte ou aconselhamento aos pais/cuidadores.

## INTRODUCTION

The beginning of complementary feeding for babies involves several doubts. Health professionals have great responsibilities as bearers of information, directly and/or indirectly influencing the decisions of parents/caregivers regarding infant feeding.

The baby-led weaning (BLW) method is an alternative approach for introducing solid foods, but it has been the subject of questioning.^1^ ^-^ ^5^ It suggests that babies from the sixth month of life onwards have motor skills to guide their own ingestion^6^ (postural balance to sit with little or no help, in addition to stability to reach, grab and bring food to their mouth)^1^ ^,^ ^6^ ^-^ ^9^ and, therefore, as long as they show adequate growth and development, they are able to start consuming food in pieces, strips or sticks, instead of porridge or purees by means of a spoon.^6^ ^,^ ^10^ In short, in the BLW method, parents/caregivers act in feeding as an intermediate, because babies themselves have the leadership not only of what and how much is eaten, but also of the speed with which they take meals.^6^ ^,^ ^10^

However, although the method is spreading among the world population, particularly in the United Kingdom, New Zealand and Canada,^1^ ^,^ ^2^ ^,^ ^4^ ^,^ ^11^ there is still no robust evidence on this practice. To date, there are only three original works in the literature addressing the perceptions of health professionals, with New Zealanders (general practitioners, lactation consultants, nurses, nutritionists, midwives, pediatricians and language therapists),^12^ Canadians (lactation nurses, nurses, physical therapists, physicians, nutritionists and occupational therapists)^11^ and Spaniards (pediatricians),^13^ whose results showed that BLW was a source of uncertainty among health professionals working in pediatrics (or similar subarea). Most of them did not feel fully convinced to recommend it because of the concern with the risk of suffocation and because they suspect that the method could have a negative impact on energy consumption and iron intake.^3^ ^,^ ^11^ ^-^ ^13^

Therefore, since the theme is unprecedented in Brazil, this exploratory study aimed to describe the perceptions of Brazilian health professionals about the practice of the BLW method for complementary feeding.

## METHOD

This is a cross-sectional study that covered Brazilian health professionals graduated in Nursing, Speech Therapy, Medicine, Nutrition or Dentistry who worked in pediatrics (or similar subarea) and who were directly or indirectly involved in child nutrition.

The sampling was non-probabilistic, of convenience, carried out by the exponential snowball technique, with adaptations.^14^ This technique was chosen because of the complexity of access to health professionals by the researchers.

Participants were, therefore, recruited through the following procedures:

- Groups or organizations/institutions of health professionals working in pediatrics (or similar subarea) were identified, in order to access those who met the criteria of the study.
- We post invitation on social media and sent contacts by e-mail and/or messaging applications, with nominal invitations issued for participation and clarifications about the objectives, inclusion criteria and confidentiality standards of the study.
- At the end of the questionnaire, health professionals were asked to: indicate two or more individuals from the same work network, but who were not limited to very close contacts, to also be invited to participate in the research; They were also asked to share the study message, which included a web link to the questionnaire.

These strategies were carried out systematically until they had no further effect on the sample size. Data collection took place from October 2018 to July 2019.

Of the 498 health professionals who agreed to participate in the study, those who had the following characteristics were excluded:

- not being directly or indirectly involved in child nutrition (n=19).
- less than one year of professional experience in pediatrics (or similar subarea) (n=11).
- not completing the questionnaire in its entirety (n=10).

Thus, 458 participants were selected.

The questionnaire was managed through the Google Forms application (www.google.com/forms/about), being self-administered by healthcare professionals, with online filling within 30 days from the date of issuance of nominal invitations. The file addressed the participants’ demographic characteristics, qualifications and workplace, in addition to knowledge, clinical practice and perceptions about possible benefits of the BLW method. The last section consisted of ten statements (Chart 1) and five categories of response on a Likert scale—totally agreed, partially agreed, indifferent (did not know, had no experience or had no clearly defined position), partially disagreed and totally disagree. The content was inspired by the study by Rubio et al.,^13^ with adaptations based on Arantes et al.,^3^ D’Andrea et al.^11^ and Cameron et al.^12^

**Chart 1:** Section of the questionnaire addressing the perceptions of health professionals about possible benefits of the baby-led weaning method. Brazil, 2018/2019.

Indicate your degree of agreement or disagreement with the following statements	
Statement A	The BLW method can make babies more likely to share family meal times
Statement B	The BLW method can facilitate babies’ adaptation to different food flavors and consistencies.
Statement C	The BLW method can enhance babies’ chewing
Statement D	The BLW method can favor the development of babies’ motor skills
Statement E	The BLW method can prevent babies from being overweight.
Statement F	The BLW method can promote self-regulation of satiety and, therefore, lesser feeding requirements for babies.
Statement G	The BLW method generally does not result in insufficient weight gain for babies.
Statement H	The BLW method generally does not result in deficiency of some nutrients for babies.
Statement I	The BLW method can be very comfortable/convenient, as there is no need to prepare special foods for babies.
Statement J	The BLW method can generate less concern or anxiety in parents/caregivers.

BLW: baby-led weaning. The statements had five categories of response on a Likert scale: totally agree, partially agree, indifferent, partially disagree and totally disagree.

Important to note that the questionnaire was designed by two researchers and then submitted to an evaluation panel of four specialists. The critical review was based on the relevance of content, the clarity/complexity of understanding, the completeness, the absence of bias and, consequently, the possibility of success. In addition, they also carried out two pre-tests to assess in order to assess the ideal ordering of the statements, the understanding of the response structure and the average time to complete.

The study was approved by the Institutional Research Ethics Committee (CAEE: 96134918.5.0000.5147; protocol number 3.191.683) and was carried out in accordance with the guidelines established in the Declaration of Helsinki, requesting the consent of the participants in a written informed consent form.

All results were shown in absolute (n) and relative (%) frequencies, aided by the software IBM Statistical Package for the Social Sciences (version 20.0, ^©^IBM Corp., United States).

## RESULTS

Table 1 shows the demographic characteristics, the titles and the place of work of health professionals. The sample (n=458) had mean age of 34.5±8.5 years, 96.5% of them being female and 64.6% working in the Southeast of Brazil. Of the total, 10.5% were graduated in Nursing, 10.3% in Speech Therapy, 12.7% in Medicine, 65.3% in Nutrition and 1.3% in Dentistry. In addition, 30.1% had between six and ten years of professional experience; 45.2, 15.3, 30.8 and 12.4% had attended or were still attending, respectively, *lato sensu* specialization in Pediatrics (or related subarea), *lato sensu* specialization in Family Health (or related subarea), master's degree (professional or academic) and doctorate. Most of them performed professional activities in clinics, ambulatorial care, outpatient clinics or home care (77.9%) in the private sector (57.4%).

**Table 1:** Demographic characteristics, qualifications and workplace of health professionals. Brazil, 2018/2019.

(n=458)		Frequency	
		n	%*
Sex			
	Female	442	96.5
	Male	16	3.5
Age (Years)			
	22-29	137	29.9
	30-39	227	49.6
	40-49	62	13.5
	50 and older	32	7.0
Administrative region of Brazil where they performed professional activities†			
	North	29	6.3
	Northeast	57	12.4
	Mid-West	15	3.3
	Southeast	296	64.6
	South	61	13.3
Professional category			
	Nursing	48	10.5
	Speech Therapy	47	10.3
	Medicine	58	12.7
	Nutrition	299	65.3
	Dentistry	6	1.3
Time of professional experience in Pediatrics (or related subarea) (years)			
	1-5	132	28.8
	6-10	138	30.1
	11-15	92	20.1
	16-20	44	9.6
	21 and more	52	11.4
Had Attended or was attending a *lato sensu* postgraduate course in Pediatrics (or related subarea)			
	Attended	164	35.8
	Attending	43	9.4
Had Attended or was attending a *lato sensu* postgraduate course in Family Health (or related subarea)			
	Attended	55	12.0
	Attending	15	3.3
Had Attended or was attending a post-graduation course (Master level)			
	Attended	121	26.4
	Attending	20	4.4
Had Attended or was attending a post-graduation course (PhD level)			
	Attended	33	7.2
	Attending	24	5.2
Type of service/institution at which they work in activities related to pediatrics (or related subarea)‡			
	Collective feeding in daycare or school	48	10.5
	Clinic, outpatient clinic or home care	357	77.9
	Other	53	11.6

* Valid percentages

† covering 137 municipalities in 24 Brazilian states

‡ 57.4% in private service.

Table 2 shows the data related to the knowledge and clinical practice of health professionals regarding the BLW method. Most of them reported knowing the method (82.1%), having as main information a course, lecture, professional meeting or seminar/symposium/congress (36.2%) and the scientific literature (32.2%). As for clinical practice, 38.3% mentioned that they used to recommend it sometimes, 37.5%, frequently, and 20.5% always.

**Table 2:** Knowledge and clinical practice of health professionals about the baby-led weaning method, according to their undergraduate course. Brazil, 2018/2019.

(n=458)		Total n (%)*	Professional category n (%)**				
			Nursing	Speech therapy	Medicine	Nutrition	Dentistry
Knew the BLW method						
	Yes	376 (82.1)	30 (8.0)	36 (9.6)	38 (10.1)	267 (71.0)	5 (1.3)
	Total	458 (100)	48 (10.5)	47 (10.3)	58 (12.7)	299 (65.3)	6 (1.3)
Main source of information about the BLW method †						
	The parents/caregivers of the babies themselves	15 (4.0)	1 (6.7)	0 (0.0)	6 (40.0)	6 (40.0)	2 (13.3)
	Any course, lecture, professional meeting or seminar/symposium/congress	136 (36.2)	6 (4.4)	15 (11.0)	6 (4.4)	109 (80.1)	0 (0.0)
	A webpage	60 (16.0)	10 (16.7)	8 (13.3)	9 (15.0)	30 (50.0)	3 (5.0)
	A professional colleague	44 (11.7)	3 (6.8)	4 (9.1)	3 (6.8)	34 (77.3)	0 (0.0)
	The scientific literature	121 (32.2)	10 (8.3)	9 (7.4)	14 (11.6)	88 (72.7)	0 (0.0)
	Total	376 (100)	30 (8.0)	36 (9.6)	38 (10.1)	267 (71.0)	5 (1.3)
Knew the benefits of the BLW method†						
	Yes	363 (96.5)	29 (8.0)	36 (9.9)	37 (10.2)	256 (70.5)	5 (1.4)
	Total	376 (100)	30 (8.0)	36 (9.6)	38 (10.1)	267 (71.0)	5 (1.3)
Used to recommend the BLW method †						
	Always	77 (20.5)	10 (13.0)	6 (7.8)	5 (6.5)	54 (70.1)	2 (2.6)
	Frequently	141 (37.5)	7 (5.0)	19 (13.5)	16 (11.3)	96 (68.1)	3 (2.1)
	Sometimes	144 (38.3)	12 (8.3)	11 (7.6)	15 (10.4)	106 (73.6)	0 (0.0)
	Never	14 (3.7)	1 (7.1)	0 (0.0)	2 (14.3)	11 (78.6)	0 (0.0)
	Total	376 (100)	30 (8.0)	36 (9.6)	38 (10.1)	267 (71.0)	5 (1.3)
Main reason for never recommending of the BLW method‡						
	Being afraid that babies could choke/asphyxiate	0	0	0	0	0	0
	Being afraid that the BLW method would result in insufficient nutritional input (energy and/or micronutrients) for babies	5 (35.7)	0	0	1 (20.0)	4 (80.0)	0
	Lack of scientific evidence	3 (21.4)	0	0	0	3 (100)	0
	Did not have satisfactory knowledge	6 (42.9)	1 (16.7)	0	1 (16.7)	4 (66.7)	0
	Total	14 (100)	1 (7.1)	---	2 (14.3)	11 (78.6)	---
Has seen the BLW method in practice†						
	Yes	296 (78.7)	23 (7.8)	32 (10.8)	27 (9.1)	209 (70.6)	5 (1.7)
	Total	30 (100)	36 (8.0)	38 (9.6)	267 (10.1)	5 (71.0)	30 (1.3)
Assisted a family who followed the BLW method†						
	Yes	192 (51.1)	9 (4.7)	22 (11.5)	22 (11.5)	137 (71.4)	2 (1.0)
	Total	30 (100)	36 (8.0)	38 (9.6)	267 (10.1)	5 (71.0)	30 (1.3)

BLW, baby-led weaning

* valid percentages per column

** valid percentages per line

† considering health professionals who knew the BLW method (n=376)

‡ considering health professionals who never recommended the practice of the BLW method (n=14).

Those who never recommended BLW (3.7%) pointed out the following reasons:

- being afraid that the method would reflect insufficient nutritional input (energy and/or micronutrients) for babies;
- lack of scientific evidence;
- not having satisfactory knowledge.

There were no reports of concern about the risk of suffocation. In addition, 78.7% of the participants had already witnessed the BLW in action and just over half (51.1%) claimed that they attended a family that use this method for complementary feeding.

**Figure 1:**
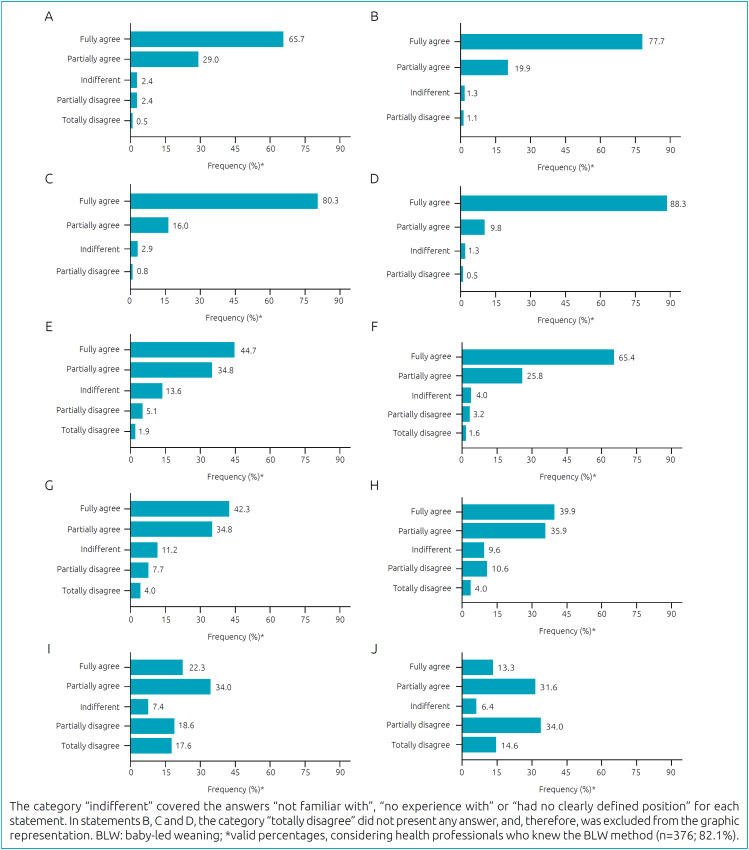
Perceptions of health professionals about possible benefits of the baby-led weaning method. Brazil, 2018/2019. (A) Statement A: the BLW method can make babies more likely to share family meal times. (B) Statement B: the BLW method can facilitate babies’ adaptation to different food flavors and consistencies. (C) Statement C: the BLW method can enhance babies’ chewing. (D) Statement D: the BLW method can favor the development of babies’ motor skills. (E) Statement E: the BLW method can prevent babies from being overweight. (F) Statement F: the BLW method can promote self-regulation of satiety and promote lesser feeding requirements for babies. (G) Statement G: the BLW method generally does not result in insufficient weight gain for babies. (H) Statement H: the BLW method generally does not result in deficiency of some nutrients for babies. (I) Statement I: the BLW method can be very comfortable/convenient, as there is no need to prepare special foods for babies. (J) Statement J: the BLW method can generate less concern or anxiety in parents/caregivers.

Figure 1 illustrates the perceptions of health professionals about possible benefits of BLW. Most of them totally agreed that the method could:

- make babies more likely to share family meals (statement A, 65.7%);
- facilitate adaptation to different flavors and consistencies of food (statement B, 77.7%);
- enhance chewing (statement C, 80.3%);
- help in the development of motor skills (statement D, 88.3%);
- encourage self-regulation of satiety and promote fewer dietary requirements (statement F, 65.4%).

However, important frequencies of partial agreement and/or disagreement were found in relation to the other parameters, especially statement I (“the BLW method can be very comfortable/convenient, as there is no need to prepare special foods for babies”), with disagreement of 36.2%, and statement J (“the BLW method can generate less concerns or anxiety in parents/caregivers”), with disagreement of 48.6%.

## DISCUSSION

This study showed that the majority of Brazilian health professionals knew the BLW and used to recommend it frequently or sometimes. There were no reports of concern about the risk of suffocation, and just over half of the sample assisted a family that followed this method. In addition, most of them stated that they totally agreed that the BLW could bring advantages for babies, however important disagreements were expressed in relation to their comfort/convenience and the argument of generating less concerns or anxiety in parents/caregivers.

Cameron et al.,^12^ after interviewing 31 New Zealand health professionals (general practitioners, lactation consultants, nurses, nutritionists, midwives, pediatricians and language therapists), reported that 41.9% of them had heard about the method. But, corroborating our study (82%), D’Andrea et al.,^11^ with 33 Canadian health professionals (lactation consultants, nurses, physiotherapists, physicians, nutritionists and occupational therapists), and Rubio et al.,^13^ with 579 Spanish pediatricians, found, respectively, that 81.8 and 79.4% knew the BLW method. We can infer that the discrepancy of Cameron et al.^12^ was due to the time when data was collected, 2010, with their research being relatively older than the other two, conducted in 2014 and 2015. In fact, this method began to gain notoriety in 2008, with the publication of the work of Rapley and Murkett entitled “Baby-led weaning: helping your baby to love good food”. To exemplify this substantial growth in popularity, in December 2016, a search on Google (www.google.com) for the baby-led weaning nomenclature returned just under a million results;^2^ in November 2019, the same search achieved nearly nine million results, covering numerous websites, blogs and online forums dedicated to sharing experiences about the method.

In our study, among the sources of information about the BLW, there were some courses, lectures, meetings or symposium/congresses (36%), and the scientific literature (32%). Canadian health professionals claimed to have become aware of the method through courses/training, patients (i.e., the babies’ parents/caregivers themselves) and professional colleagues.^11^ Spanish pediatricians also revealed that their main sources of information were a course or lecture (29.2%) and the mothers of the babies (21.2%).^13^

Some studies have found that a large number of parents/caregivers were presented the BLW in groups of parents, friends or in a web page.^11^ ^,^ ^12^ ^,^ ^15^ It was also reported that mothers who adhere to the method, compared to those who follow the traditional complementary diet (with porridge or purees by means of a spoon), turned less to the support of pediatricians.^12^ ^,^ ^15^ Therefore, such results brings about a certain lack of information about the method among health professionals, something that supposedly has been changing in the past years, although there are no updated population surveys.

In our study, just over half of the participants (50.9%) assisted a family that followed the BLW method, a frequency very close to that of Spaniard pediatricians (49.9%),^13^ which, once again, highlights the increasing popularity of this method.

As for the clinical practice of BLW, a larger number of our participants used to recommend it (38.1%, sometimes; 37.6%, often; and 20.6%, always), compared to Spaniard pediatricians (45.3%, sometimes; and 6.6%, always).^13^ Diverging from the literature,^11^ ^-^ ^13^ Brazilian health professionals did not express concern about the risk of suffocation. Although some studies have concluded that there were no differences in the frequency of asphyxiation episodes between groups of babies adhering to BLW and traditional complementary feeding,^16^ ^-^ ^19^ caution and more robust evidence is needed.^4^

It is relevant to say that New Zealand and Canadian health professionals were strongly concerned about the possibility of the method resulting in energy and iron deficits, thus impairing the growth and development of babies,^11^ ^,^ ^12^ but only 5.2 and 1.4% of Spaniard pediatricians cited, in this order, the low energy contribution and the risk of iron deficiency as reasons for not indicating it.^13^

Paying attention to the concerns mostly mentioned in the literature, in 2015, researchers proposed a modified version of the BLW, called baby-led introduction to solids (BLISS), in which 12 recommendations were outlined, aiming at the prevention of risk of choking, low energy consumption and iron deficit.^20^ The difference between BLW and BLISS is restricted to these instructions, while the main characteristics remain the same.^20^ ^,^ ^21^

Recently, randomized controlled clinical trials have reported that babies introduced to BLISS, compared with those who were exposed to a traditional complementary feeding, were not more susceptible to episodes of asphyxia,^18^ or to inadequacies in the consumption of energy and micronutrients^22^ ^-^ ^25^ and in iron^24^ and zinc intake.^25^ Taylor et al.^22^ also found out that there were no differences in the z-scores of the body mass index for babies in range of 12 and 24 months of age. Despite this, they did not exclude the chance of a potentially significant increase in the risk of being overweight.

Regarding the perceptions about possible benefits of the BLW method, the aspects commonly valued by health professionals in Brazil, New Zealand, Canada and Spain were encouragement to share family meals, encouragement of chewing and promotion of development of motor skills.^11^ ^-^ ^13^ Corroborating our study, Canadians also stated that the method could favor self-regulation of satiety and promote less dietary demands,^11^ while the comfort/convenience and the argument of generating less concern or anxiety in parents/caregivers, which represented the main targets of disagreement among Brazilian health professionals, were pointed out as advantages by New Zealanders and Canadians.^11^ ^,^ ^12^ As for the statements about preventing overweight and not resulting in nutritional deficiencies, Brazilians and Spaniards were divided, with important frequencies of partial agreement and/or disagreement.^13^

Overall, the literature has an emerging, however small, body of evidence about the BLW. Furthermore, no original study has been carried out with Brazilian babies and parents/caregivers, which restricts the extrapolation of certain findings, since the practice of the method, which involves the act of eating in all its essence, is the result of a series of socio-cultural determinants more than any strictly biological parameter. There are many issues not fully clarified: the risk of suffocation, sensitization and allergic reactions to food, the effects on growth and development and the adequacy of nutrient intake (especially iron and vitamins or trace elements).^2^ ^,^ ^4^

In 2017, the Brazilian Society of Pediatrics (SBP) commented on the BLW method in a practical update guide,^26^ emphasizing its official guidelines (revised and expanded in 2018),^27^ which advocate the provision of purees at the beginning of complementary feeding, with the consistency evolving gradually until reaching the family's diet, according to the neuropsychomotor development rate of each child. In the document, the SBP further clarified: “The infant can receive the mashed food offered in the spoon, but they should also experiment with their hands, explore different textures of food as a natural part of sensory motor learning”.^26^

In line with these recommendations, in 2019, the new Food Guide for Brazilian children under the age of 2^28^ signaled, among 12 steps to healthy eating, that it is necessary to “offer mashed food when the child starts eating foods other than breast milk” and that “one should evolve into foods chopped into small pieces, scraped or shredded to learn to chew them. Soft foods can also be offered in large pieces so that the child can take them by hand and bring them to their mouth. When older enough, the child can eat the family food, cutting the large pieces when necessary.”^28^

These two official documents converge on recommendations. As there is still no scientific evidence enough and with satisfactory quality to affirm that the BLW method is the most correct form of food introduction, the traditional approach is still priority, with possibility of introducing in the method the encouragement to explore different foods and textures with their hands.^26^ ^-^ ^28^

Although this study is the first to describe the perceptions of Brazilian health professionals about the practice of the BLW method for complementary feeding, there are some limitations:

- Non-probabilistic sampling, carried out by the snowball technique, which does not guarantee representativeness or allow estimating statistical power (the final sample was not sufficiently diverse, since more than half of participants worked in the Southeast of the country and were graduated in Nutrition); however; the analysis of representative data did not figure as something fundamental because of the exploratory nature of the study.
- The evaluation of the perceptions of health professionals involved a questionnaire that has not yet been validated, but it is an instrument that has theoretical and scientific support^3^ ^,^ ^11^ ^-^ ^13^ and was elaborated with rigor, with critical review by a committee of experts and two pre-tests.
- The fact that the questionnaire was sent by e-mail and/or messaging applications does not allow to understand the circumstances in which it was completed; however, it has been demonstrated that research results obtained through the web are consistent with traditional methods of data collection.^29^

In conclusion, although many participants fully agreed that the BLW could be advantageous (making babies more likely to share family meals, facilitating adaptation to food flavors and consistencies, enhancing chewing, favoring the development of motor skills and self-regulation of satiety, and promoting less dietary requirements), there were important frequencies of partial agreement (in relation to statements about preventing excess weight and not resulting in nutritional deficiencies) and disagreement (regarding comfort/convenience and the argument of generating less concern or anxiety in parents/caregivers), which is likely a reflection of the scarcity of factual scientific evidence on this topic. Given the increasing popularity of the BLW method, there is an urgent need for further studies to better understand risks and benefits in different contexts and populations. Only then will health professionals working in pediatrics (or a related sub-area) be able to choose the most appropriate method and feel effectively informed to provide support or advice to parents/caregivers.
